# A Cross-Sectional and Longitudinal Integrated Study on Brain Functional Changes in a Neuropathic Pain Rat Model

**DOI:** 10.1523/ENEURO.0272-23.2024

**Published:** 2024-03-06

**Authors:** Xin-Tian Chi, Wu Yang, Jian-Bo Zhang, Yu-Tao Lei, Chen-Chen Tao, Hong-Ni Chen, Zi-Kun Zheng, Wen-Jun Xin, Ting Xu, Shuang Gao, Xue-Qin Zhang

**Affiliations:** ^1^The Affiliated Brain Hospital of Guangzhou Medical University (Guangzhou Huiai Hospital), School of Health Management and Institute of Mental Psychology, Guangzhou Medical University, Guangzhou 511495, China; ^2^Department of Pain Management, State Key Specialty in Pain Medicine, Guangzhou Medical University Second Affiliated Hospital, Guangzhou 510260, China; ^3^Department of Electronic Engineering, Shantou University, Shantou 515063, China; ^4^Guangdong Province Key Laboratory of Brain Function and Disease, Department of Physiology, Zhongshan School of Medicine, Sun Yat-Sen University, Guangzhou 510080, China

**Keywords:** functional connectivity, low-frequency fluctuations, neuropathic pain, regional homogeneity, resting-state functional magnetic resonance imaging

## Abstract

Human and animal imaging studies demonstrated that chronic pain profoundly alters the structure and the functionality of several brain regions and even causes mental dysfunctions such as depression and anxiety disorders. In this article, we conducted a multimodal study cross-sectionally and longitudinally, to evaluate how neuropathic pain affects the brain. Using the spared nerve injury (SNI) model which promotes long-lasting mechanical allodynia, results showed that neuropathic pain deeply modified the intrinsic organization of the brain functional network 2 weeks after injury. There are significant changes in the activity of the left thalamus (Th_L) and left olfactory bulb (OB_L) brain regions after SNI, as evidenced by both the blood oxygen level-dependent (BOLD) signal and c-Fos expression. Importantly, these changes were closely related to mechanical pain behavior of rats. However, it is worth noting that after morphine administration for analgesia, only the increased activity in the TH region is reversed, while the decreased activity in the OB region becomes more prominent. Functional connectivity (FC) and c-Fos correlation analysis further showed these two regions of interest (ROIs) exhibit different FC patterns with other brain regions. Our study comprehensively revealed the adaptive changes of brain neural networks induced by nerve injury in both cross-sectional and longitudinal dimensions and emphasized the abnormal activity and FC of Th_L and OB_L in the pathological condition. It provides reliable assistance in exploring the intricate mechanisms of diseases.

## Significance Statement

Neuropathic pain is a debilitating condition caused by lesion or disease of the somatosensory nervous system. In the present study, we applied a noninvasive neuroimaging technique, functional magnetic resonance imaging (fMRI) to elucidate the underlying mechanisms. We showed the significant changes in brain activity and functional network connectivity in rats following nerve injury through the simultaneous implementation of both cross-sectional and longitudinal research. The thalamus and olfactory bulb were further identified as the key functional brain regions that might be involved in SNI-induced mechanical hypersensitivity, which provides reliable ideas and directions for further exploring the brain network and neural circuit mechanisms of neuropathic pain.

## Introduction

Neuropathic pain is a common chronic pain condition caused by lesion or disease of the somatosensory nervous system that may paradoxically lead not only to loss of function but also to increased pain sensitivity and spontaneous pain (Scholz et al., 2019). The prevalence of neuropathic pain is between 6.9 and 10% of the general population (van Hecke et al., 2014). The global burdens of disease associated with chronic pain are in many cases increasing (Rice et al., 2016). Furthermore, the pathogenic factors of neuropathic pain are numerous, and its underlying mechanism is still unclear. Animal models have provided reliable assistance in exploring the intricate mechanisms of diseases. Including our own work, the spared nerve injury (SNI) model has been largely characterized in terms of behavioral and functional alterations; it demonstrates substantial and prolonged changes in behavioral measures of mechanical sensitivity and has proved to be a robust model of neuropathic pain; and it is a tool for identifying pathophysiological mechanisms of neuropathic pain and testing pharmacological agent (Challa, 2015; Guida et al., 2020; Zhao et al., 2022).

Functional magnetic resonance imaging (fMRI) is a noninvasive neuroimaging technique that is uniquely magnetically sensitive to oxygen-rich blood and that has been widely used to reflect the spontaneous activity of the brain through changes in blood oxygen level-dependent (BOLD) signals (Smith, 2012). Furthermore, resting-state fMRI (rs-fMRI) aims to macroscopically detect resting-state neuronal activity in response to the possible role of resting-state–specific sustained experience in shaping functional connectivity (FC; Smitha et al., 2017; Sezer et al., 2022). Both the amplitude of low-frequency fluctuations (ALFF), regional homogeneity (ReHo), and seed-based FC are reliable scan indicators in rs-fMRI (Zhao et al., 2018). The ALFF signals represent the response of individual voxels, characterizing spontaneous neural activity in local brain region, and the ReHo calculates the synchronization of low-frequency fluctuations between a given voxel with neighboring voxels, reflecting the neural function synchronization in local brain region. The intersection of the voxels calculated by ALFF and ReHo reflects the regions that not only show activity at the same temporal frequency but also synchronize with adjacent voxels. In other words, these regions represent relatively large groups of synchronously active neurons (Lv et al., 2018). The FC is the temporal correlation of neuronal activity between separate brain regions. As is well known, the intrinsic FC of brain networked structures is disrupted in a number of nerves system diseases including chronic pain (Ceko et al., 2015; Arnold Anteraper et al., 2019; Pico-Perez et al., 2019). FC is exactly an indicator that can effectively reflect changes in connectivity of brain regions, providing a reliable target for us to study brain network changes in depth.

Although a small number of studies on the effects of SNI-induced neuropathic pain on rs-fMRI indicators in rats have been published so far, for example: a longitudinal study found that the functional characteristics of the nucleus accumbens undergo remodeling after SNI (Chang et al., 2014); another longitudinal study found that both functional metrics and connectivity of the part A of the retrosplenial granular cortex were significantly correlated with the development of neuropathic pain behaviors and associated emotional alterations (Barriere et al., 2019). They confirmed that fMRI is a noninvasive, feasible, and efficient way to study the changes in brain networks associated with neuropathic pain and also provide corresponding potential target brain regions related to disease. Considering that longitudinal studies could eliminate factors such as genetic, hereditary, and gender-based influences on behavior and MRI results between individual organisms, and cross-sectional studies could exclude factors such as age and anesthesia frequency from affecting experimental results. In present study, we adopted a pattern of cross-sectional and longitudinal research to investigate changes in brain regions and functional connections related to neuropathic pain. This can further enhance potential statistical power and increase the credibility of the results.

## Materials and Methods

### Animals and experimental design

Male Sprague Dawley (SD) rats (7–8 weeks old, 200–250 g) were obtained from the Institute of Experimental Animals. Rats were housed in cages (3–4 rats per cage) with controlled ambient temperature (22–24°C) and humidity (40–60%) on a 12 h day/dark schedule. Food and water were freely provided, except during the restraint procedure. All experimental procedures were approved by the Local Animal Care Committee and were performed in accordance with the guidelines of the National Institutes of Health on animal care and the ethical guidelines.

**Figure 1.**
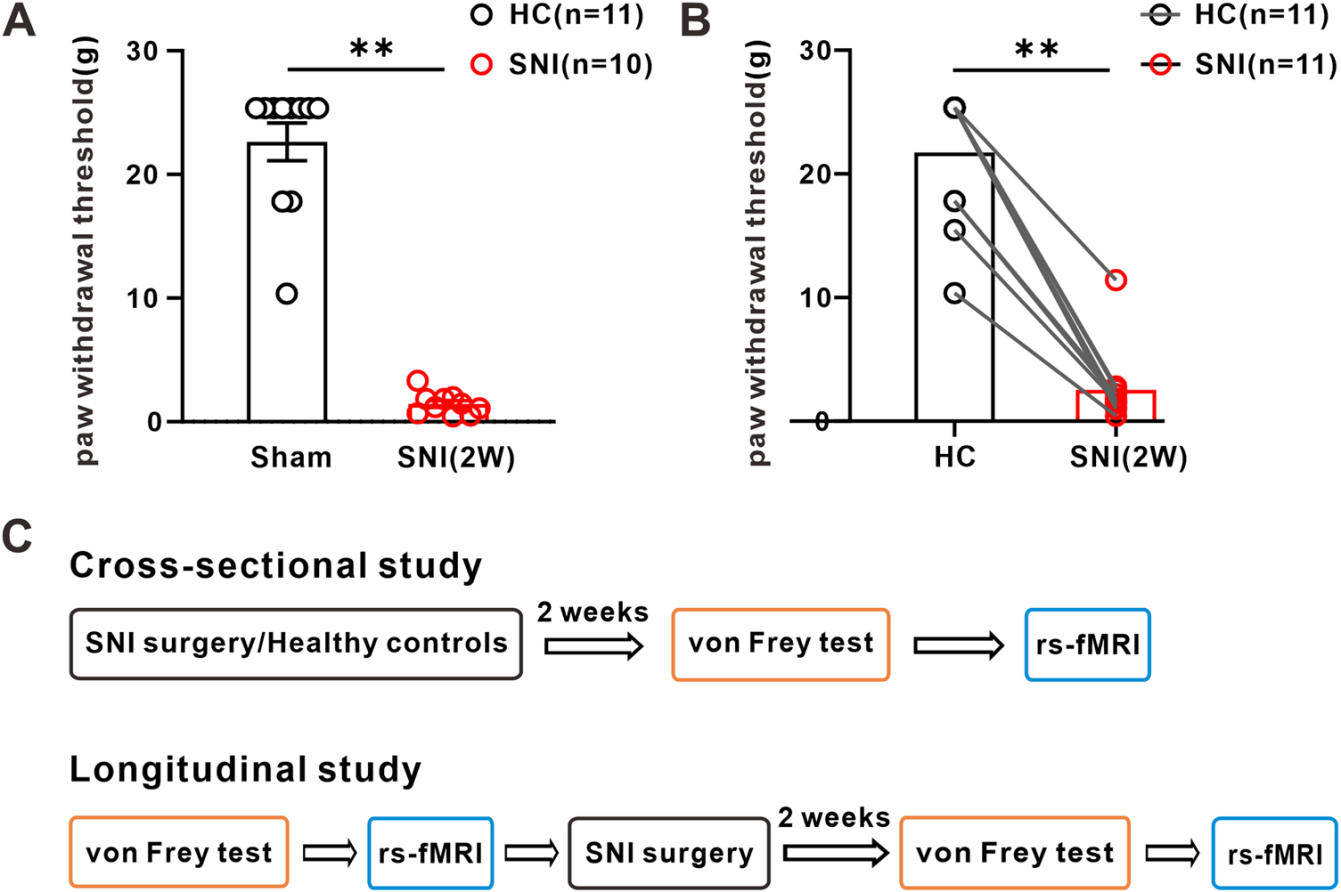
SNI-induced mechanical allodynia in rats and the schematic diagram of study. ***A***, Compared with that of sham, the paw withdrawal threshold of the left (ipsilateral of surgery) hindpaw of rats decreased significantly 2 weeks after SNI (sham group, *n* = 11; SNI group, *n* = 10; *t* test; **p < 0.01 vs the corresponding control group). ***B***, The paw withdrawal threshold of the left hindpaw of rats was significantly decreased after SNI surgery compared with that of their own controls (*n* = 11; paired *t* test; **p < 0.01 vs the corresponding control group). ***C***, Schematic diagram of cross-sectional and longitudinal experimental design and process.

All rats were randomly assigned to cross-sectional study and longitudinal study. And all animals were handled for 5 min every day for consecutive 3 weeks (1 week before the official experiment begins). The specific experimental design and procedures are presented in Figure 1*C*. Briefly, in the cross-sectional study, the rats in the neuropathic pain group (*n* = 10) and the control group (*n* = 11) underwent SNI and sham surgery respectively. After 2 weeks, the behavioral test conducted first, followed by rs-fMRI scanning. In the longitudinal study (*n* = 11), the rats underwent one behavioral test and rs-fMRI scanning first, followed by SNI surgery. Two weeks after the surgery, another behavioral test and rs-fMRI scan were carried out. During the studies, mechanical allodynia was assessed the day before each fMRI scan.

### SNI model

In rats under isoflurane (4%) anesthesia, the tibial and common peroneal branches of the sciatic nerve were ligated and sectioned distal to the ligation, removing 2 mm of the distal nerve stump. In sham-operated rats, the tibial and common peroneal branches of the sciatic nerve were identically exposed without ligation. The above surgical procedure was done according to the description of Decosterd and Woolf (2000).

### Behavioral test and drug administration

For behavioral test, all the experiments were conducted in a double-blind manner. Von Frey hairs were used to test the mechanical allodynia. Before the test, each animal was allowed to adapt the test environment by being placed individually in a plastic box for 3 consecutive days (15 min/day). Von Frey hairs of different strengths were used alternately to stimulate the lateral part of the plantar surface of left hindpaw (the ipsilateral side of SNI). A nociceptive response was defined as a brisk paw withdrawal or paw flinching following von Frey filament application. If no paw retraction response occurred, the next stronger fiber was selected for stimulation; if a paw retraction response was observed, the weaker stimulus was selected. The 50% paw withdrawal threshold was calculated for each animal following a previous validated updown procedure (Chaplan et al., 1994).

Morphine hydrochloride dissolved in saline to a concentration of 6 mg/ml was subcutaneously injected at a dose of 6 mg/kg. 60 min after morphine administration, and the rats were taken to the behavioral test.

### Immunofluorescence

Rats were anesthetized and transcardially perfused with 0.9% saline followed by 4% paraformaldehyde (PFA). Brains were postfixed in 4% PFA at 4°C for 12 h and then transferred to 20% sucrose for 1 d and 30% sucrose for 2–3 d. Then, brains were sectioned into 30-μm-thick slices. For immunofluorescence staining, each slice was washed three times in phosphate-buffered saline (PBS) and then blocked for 1 h in 0.3% Triton X-100 and 3% (w/v) normal donkey serum. Sections were then incubated in primary antibodies overnight at 4°C. After rinsing in PBS with Tween 20 twice and in PBS twice, the slices were incubated with fluorescence-conjugated secondary antibody at room temperature for 1.5 h. Last, slices were coverslipped on antiquenching mounting medium (Beyotime). Then, a Nikon (Eclipse Ni-E, Nikon) fluorescence microscope was used to examine the sections, and a Nikon DS-Qi2 camera was used to capture images. For primary antibodies, we used antibodies against c-Fos (rabbit, 1:400, Cell Signaling Technology, No. 2250). For the secondary antibody, we used Alexa Fluor 488 (rabbit, 1:500, Huabio, HA1121).

### rs-fMRI acquisition

All in vivo fMRI scanning took place in the morning (9:00–12:00 A.M.), when corticosteroid levels are relatively low (Dhabhar et al., 1993). The rat was preanaesthetized using isoflurane in an induction chamber (4% isoflurane in medical air). Once fully anaesthetized, the animal was transferred to a heated MR-compatible stereotactic holder which immobilized with earplugs and a tooth holder. During the scanning, anesthesia was maintained with a nose cone (1.5% isoflurane in medical air).

rs-fMRI data was collected on a 9.4 T animal MRI scanner (Bruker BioSpin) with use of a volume coil T12054V3 (for signal excitation) and a surface coil T20011V3 (for signal reception). A series of BOLD images was acquired, under 1–1.5% isoflurane (to keep ∼70 breaths/min for rats); a level of anesthesia at which the coherence of low-frequency BOLD signal fluctuations between functionally connected regions has been shown to be preserved. During imaging scan, anatomical images were first acquired using a rapid acquisition with relaxation enhancement (RARE) sequence [repetition time (TR), 5,081.564 ms; echo time (TE), 21.59 ms; matrix size, 150 × 105; field of view (FOV), 3.0 × 2.1 cm; slice number, 70; slice thickness, 0.4 mm; slice gap, 0; and resolution, 0.20 × 0.20 × 0.40 mm). fMRI data were obtained using a 2D multislice, single-shot, gradient-echo EPI sequence; for BOLD, the parameters are as follows: TR, 2,000 ms; TE, 10.332 ms; flip angle, 90°; matrix size, 100 × 70; FOV, 3.0 × 2.1 cm; slice number, 45; slice thickness, 0.6 mm; slice gap, 0; and resolution, 0.30 × 0.30 × 0.60 mm; 150 BOLD images.

### rs-fMRI data preprocessing

The resting-state MRI data were preprocessed using the SPM12 software (Statistical Parametric Mapping 12; http://www.fil.ion.ucl.ac.uk/spm). To ensure steady-state longitudinal magnetization, we removed the first five volumes of each fMRI scan. The remaining volumes were processed using the following steps: (1) voxel magnification, (2) slice timing correction, (3) realignment, (4) origin correction, and (5) coregistration to echo-planar imaging (EPI) templates before zoomed and resliced at a resolution of 3 × 3 × 3 mm using interpolation, spatial smoothing using Gaussian kernel with full width half-maximum (FWHM) 6 mm, and linear detrending. The preprocessed data was performed using linear detrends and temporal bandpass filtering (0.01–0.1 Hz). rs-fMRI volumes with relative framewise displacement (FD) >0.3 mm were excluded.

ALFF and ReHo values were calculated using DPABI (http://rfmri.org/dpabi) and band filtered (0.01–0.1 Hz) to reduce noise. ALFF and ReHo values for each voxel were normalized by *z*-score transformation in order to normalize the variation at the population level while taking into account interindividual differences in mean signal intensity. Statistical analysis is based on zALFF and zReHo for each voxel, and statistically significant clusters of zALFF and zReHo signals are mapped to the rat SIGMA atlas for presentation via xjView (https://www.alivelearn.net/xjview/).

FC analysis was conducted using seed-based correlational analysis. Brain regions of interest (ROIs) were extracted using DPABI from the rat SIGMA atlas based on the intersection of ALFF and ReHo results. Then, Pearson’s correlation between the time course of each seed and the time course of other voxels were calculated to obtain individual FC maps. Based on the intersection of cross-sectional and longitudinal results, taking the longitudinal results for the 3D representation of the rat brain, all data were obtained using the BrainNet Viewer (https://www.nitrc.org/projects/bnv/).

### Statistical analysis

Statistical analyses were performed using SPSS 25 (IBM). Behavioral data were presented as mean ± standard error (SEM). SPM12 (Statistical Parametric Mapping, http://www.fil.ion.ucl.ac.uk/spm/software/spm12) was used for fMRI data. The differences between sham and SNI rats in cross-sectional study were assessed using the unpaired two-sample *t* test, and differences between SNI and their own baseline in the longitudinal study were assessed using the paired *t* test. Prior to analyses, we checked the conformance to the normal distribution by using the Shapiro–Wilk normality test, and the Wilcoxon rank-sum test would be used when normal distribution was not supported. The correlation was measured by Pearson’s correlation analysis. A *p* < 0.05 was considered statistically significant. The MRI data results were corrected by false discovery rate (FDR) to reduce false positive results.

## Results

### SNI induces mechanical allodynia in rats

SNI is a universal and stable model for establishing neuropathic pain in rats (Decosterd and Woolf, 2000; Guida et al., 2020). In our study, rats were subjected to von Frey filament test to detect the level of mechanical allodynia. The results showed that compared with the rats in sham group the mechanical withdrawal threshold significantly decreased in SNI group (2 weeks after surgery) on the left hindlimb (cross-sectional study). A longitudinal study we conducted also showed that compared with their own baseline, SNI significantly decreased the mechanical withdrawal threshold of rats (Fig. 1*A*,*B*). The schematic diagram showed the experimental process of cross-sectional and longitudinal study (Fig. 1*C*).

### SNI induces pain-related changes in ALFF of BOLD-fMRI signals in the brain of rats

**Figure 2.**
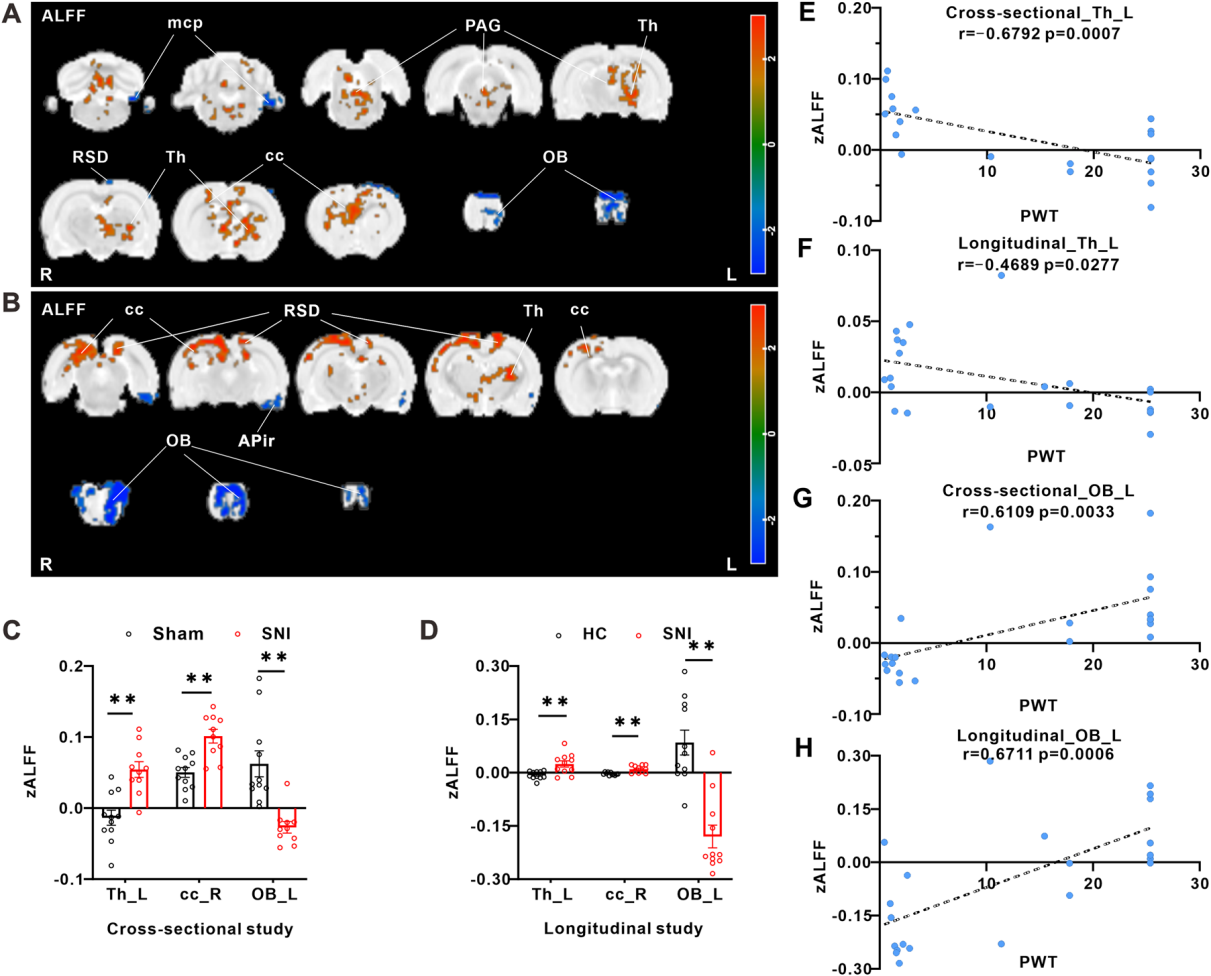
SNI-induced changes of ALFF values in different brain regions and correlations between ALFF signals and behavioral data. ***A***, Alterations of ALFF value between SNI group and sham group (bar in the right: *t* value of cluster; red represents increase and blue represents decrease; sham group, *n* = 11; SNI group, *n* = 10; *t* test; *p* < 0.05). ***B***, Alterations of ALFF value between SNI rats and their own baseline (bar in the right: *t* value of cluster; red represents increase and blue represents decrease; *n* = 11; paired *t* test; *p* < 0.05). Parameters of the intersecting brain regions of cross-sectional (***C***) study and longitudinal study (***D***) (**p < 0.01 vs the corresponding control group). ***E–H***, Significant correlations between the ALFF signals of ROIs (*y*-axis) and paw withdraw threshold (*x*-axis) in both cross-sectional (sham group, *n* = 11; SNI group *n* = 10) and longitudinal (*n* = 11) studies. R, right; L, left.

For cross-sectional study, in comparison with sham group, SNI group showed increased ALFF values in the left thalamus (Th_L), right periaqueductal gray (PAG_R), and right corpus callosum and associated subcortical white matter (cc_R), while the decreased ALFF values in the left olfactory bulb (OB_L), left retrosplenial dysgranular cortex (RSD_L), and left middle cerebellar peduncle (mcp_L; Fig. 2*A*). The local ALFF values obtained by two-sample *t* test (*p* < 0.05) are listed in Table 1.

**Table 1.** Results of ALFF analysis of cross-sectional study

	Brain regions	MNI coordinates			Voxel size	Peak
		*x*	*y*	*z*		
Pain > HC	Periaqueductal gray R	1	−128.05	21.2	38	4.1852
	Corpus callosum and associated subcortical white matter R	4	0.95	6.2	124	5.4164
	Thalamus L	−20	−29.05	12.2	70	4.2153
Pain < HC	Middle cerebellar peduncle L	−41	−128.05	0.199	5	−4.2153
	Olfactory bulb L	4	105.95	33.2	221	−5.358
	Retrosplenial dysgranular cortex L	−32	12.95	63.2	24	−4.169

For longitudinal study, in comparison with their own control group, SNI induced the increase of ALFF values in the Th_L, cc_R, and RSD_L, while the decreased ALFF values in the OB_L and left amygdalopiriform cortex (APir_L; Fig. 2*B*). The local ALFF values obtained by two-sample paired *t* test (*p* < 0.05) are listed in Table 2. The areas with consistent changes both in cross-sectional study and longitudinal study were Th_L, cc_R (increased after SNI), and OB_L (decreased after SNI; Fig. 2*C*,*D*).

**Table 2.** Results of ALFF analysis of longitudinal study

	Brain regions	MNI coordinates			Voxel size	Peak
		*x*	*y*	*z*		
Pain > HC	Thalamus L	10	−35.05	−11.8	113	5.8116
	Corpus callosum and associated subcortical white matter R	49	−53.05	57.2	56	5.9961
	Retrosplenial dysgranular cortex L	−14	−35.05	66.2	131	5.8139
Pain < HC	Amygdalopiriform cortex L	−59	−95.05	30.2	60	−4.4194
	Olfactory bulb L	−11	60.95	24.2	1,039	−12.4662

Based on the above results, we further investigated the specific changes of Th_L and OB_L correlated with the behavioral data. A data-driven correlational analysis was conducted for these two ROIs between their average value of ALFF and the paw withdrawal threshold in painful behavioral tests. The results showed that, in both cross-sectional and longitudinal studies, there was significant negative correlation between zALFF value of Th_L and paw withdrawal threshold (Fig. 2*E*,*F*). On the contrary, in both cross-sectional and longitudinal studies, there was significant positive correlation between zALFF value of OB_L and paw withdrawal threshold (Fig. 2*G*,*H*).

### SNI induces pain-related changes in ReHo of BOLD-fMRI signals in the brain of rats

**Figure 3.**
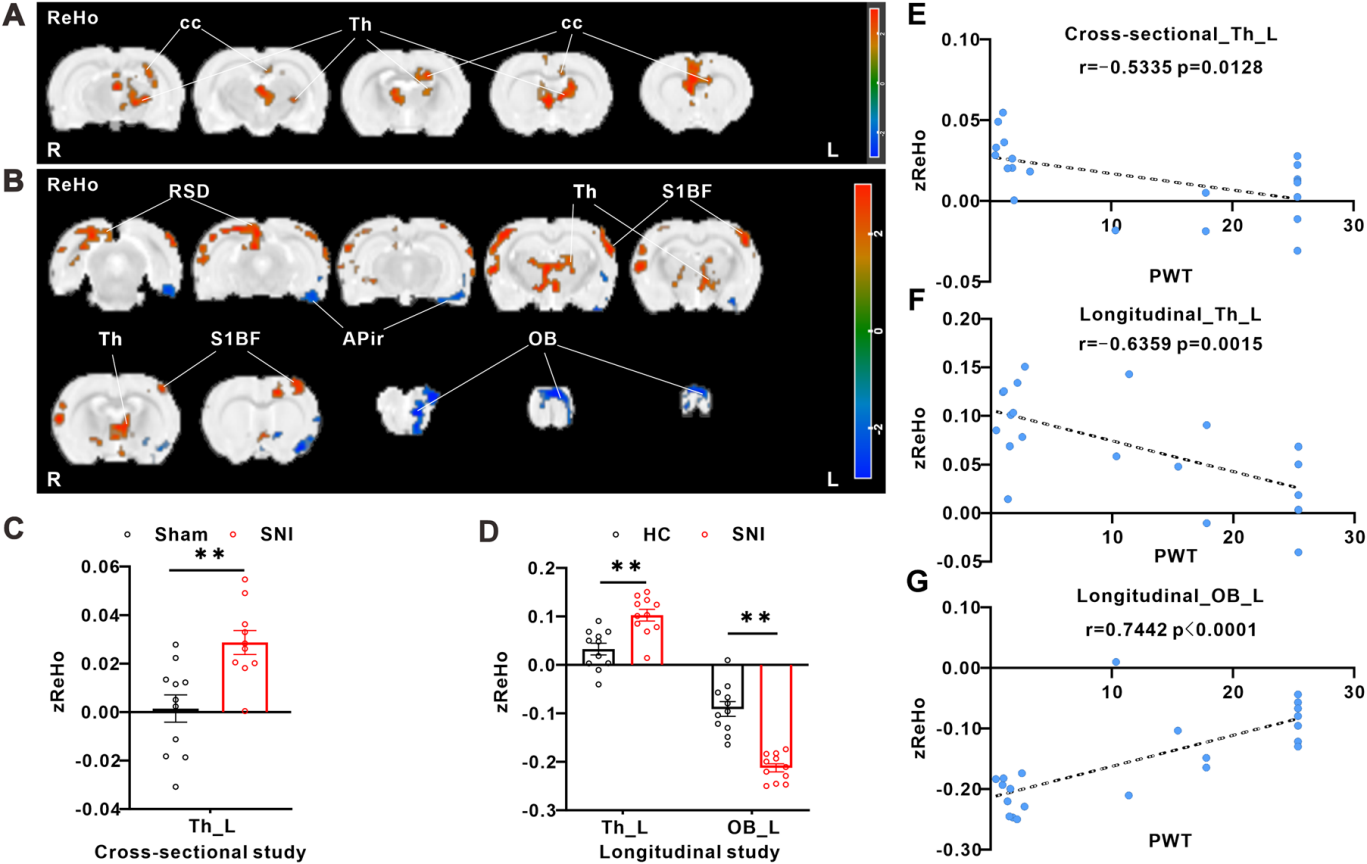
SNI-induced changes of ReHo values in different brain regions and correlations between ReHo signals and behavioral data. ***A***, Alterations of ReHo value between SNI group and sham group (bar in the right: *t* value of cluster; red represents increase and blue represents decrease; sham group, *n* = 11; SNI group, *n* = 10; *t* test; *p* < 0.05). ***B***, Alterations of ALFF value between SNI rats and their own baseline (bar in the right: *t* value of cluster; red represents increase and blue represents decrease; *n* = 11; paired *t* test; *p* < 0.05). Parameters of the intersecting brain regions of cross-sectional (***C***) study and longitudinal study (***D***; **p < 0.01 vs the corresponding control group). ***E–G***, Significant correlations between the ReHo signals of ROIs (*y*-axis) and paw withdraw threshold (*x*-axis) in both cross-sectional (sham group, *n* = 11; SNI group, *n* = 10) and longitudinal (*n* = 11) studies. R, right; L, left.

For cross-sectional study, in comparison with sham group, SNI group showed increased ReHo values in the Th_L and cc_R. There were no significantly decreased ReHo values in the whole brain (Fig. 3*A*). The local ReHo values obtained by two-sample *t* test (*p* < 0.05) are listed in Table 3.

**Table 3.** Results of regional homogeneity analysis of cross-sectional study

	Brain regions	MNI coordinates			Voxel size	Peak
		*x*	*y*	*z*		
Pain > HC	Thalamus L	−2	−47.05	24.2	65	4.5293
	Corpus callosum and associated subcortical white matter L	4	−2.05	6.2	36	5.1235

For longitudinal study, in comparison with their own control group, SNI increased ReHo values in the Th_L, right retrosplenial dysgranular cortex (RSD_R), and left primary somatosensory cortex barrel field (S1BF_L), while SNI decreased ReHo values in the OB_L and APir_L (Fig. 3*B*). The local ReHo values obtained by two-sample paired *t* test (*p* < 0.05) are listed in Table 4. The area with consistent changes both in cross-sectional study and longitudinal study was Th_L (increased after SNI; Fig. 3*C*,*D*).

**Table 4.** Results of regional homogeneity analysis of longitudinal study

	Brain regions	MNI coordinates			Voxel size	Peak
		*x*	*y*	*z*		
Pain > HC	Thalamus L	−5	−2.05	−17.8	202	5.4725
	Retrosplenial dysgranular cortex R	52	−29.05	54.2	105	6.6578
	Primary somatosensory cortex barrel field L	−41	9.95	60.2	179	7.009
Pain < HC	Amygdalopiriform cortex L	−65	−89.05	9.2	77	−4.6107
	Olfactory bulb L	−38	27.95	15.2	603	−8.9785

Based on the above results, the change of Th_L and OB_L was stable and remarkable. The correlation analysis results of average value of Reho and paw withdrawal threshold showed that in both cross-sectional and longitudinal studies, the zReHo value of Th_L was significantly negatively correlated with the mechanical paw withdrawal threshold (Fig. 3*E*,*F*). However, the zReHo value of OB_L was significantly negatively correlated with the mechanical paw withdrawal threshold in longitudinal study (Fig. 3*G*).

### Validation of activity changes in the thalamus and olfactory bulb following SNI and morphine

**Figure 4.**
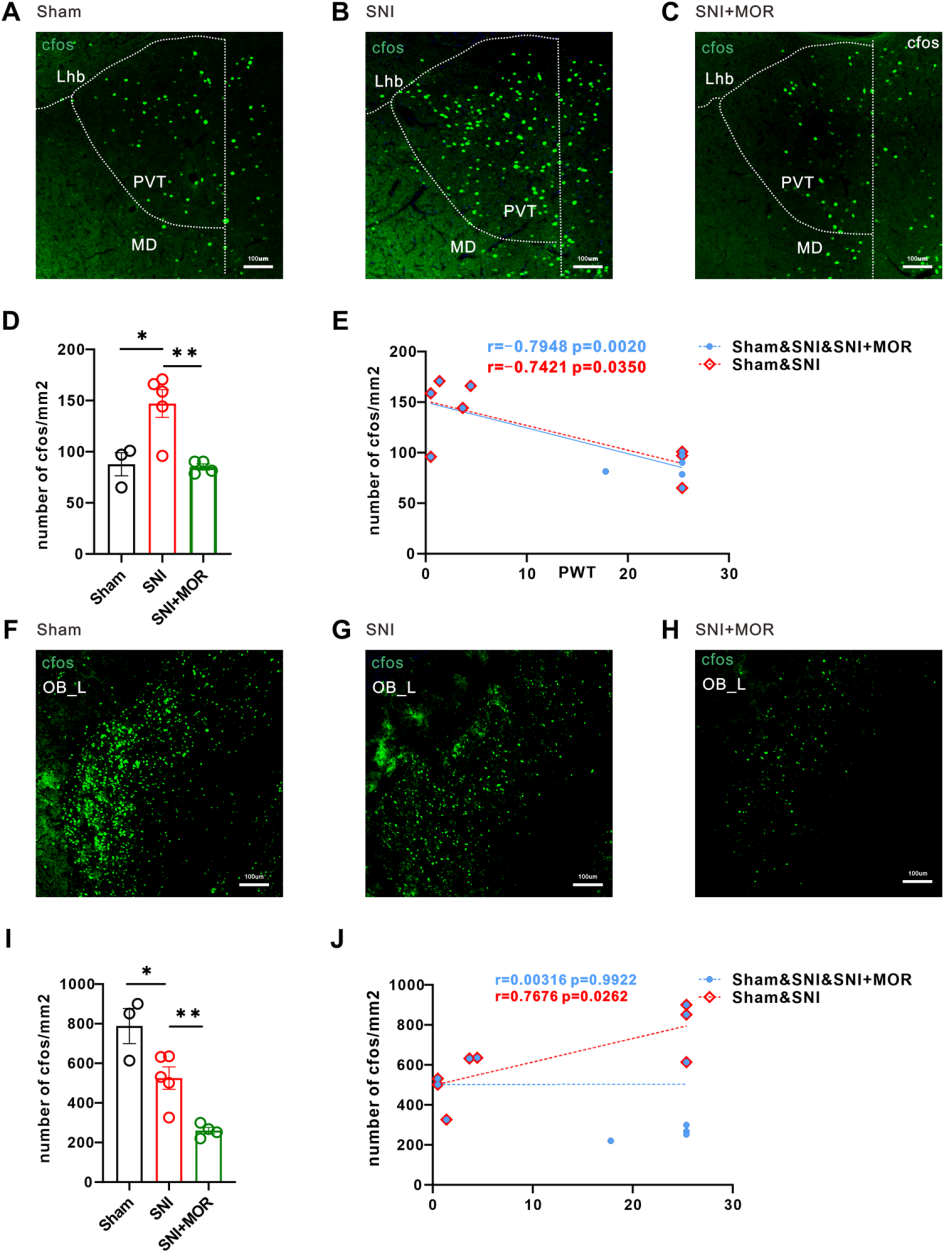
Changes of c-*fos* numbers in left thalamus and left olfactory bulb. ***A–C***, Representative immunofluorescence images of c-*fos* expression in the sham, SNI, and SNI + morphine group (scale bar, 100 μm). ***D***, Comparison of the number of c-*fos* in the sham, SNI, and SNI + MOR groups (sham group, *n* = 3; SNI group, *n* = 5; SNI + MOR group, *n* = 4; *t* test; *p < 0.05; **p < 0.01). ***E***, Correlations between changes in c-*fos* numbers of PVT_L and painful behavior (Sham&SNI&SNI + MOR, *n* = 12, blue icon; sham&SNI, *n* = 8, red icon). ***F–H***, Representative immunofluorescence images of c-*fos* expression in the sham, SNI, and SNI + morphine group (scale bar, 100 μm). ***I***, Comparison of the number of c-*fos* in the sham, SNI, and SNI + MOR groups (sham group, *n* = 3; SNI group, *n* = 5; SNI + MOR group, *n* = 4; *t* test; *p < 0.05; **p < 0.01). ***J***, Correlations between changes in c-*fos* numbers of OB_L and painful behavior (Sham&SNI&SNI + MOR, *n* = 12, blue icon; Sham&SNI, *n* = 8, red icon).

Although there was no significant downregulation of ReHo signal in OB_L in the cross-sectional study, given its significant changes in the longitudinal study and ALFF value calculation, we included OB in the subsequent analysis. To further confirm the imaging results, we performed whole-brain c-Fos staining in rats. The results revealed that compared with the sham group, the SNI group exhibited a significant increase of c-Fos expression in the Th_L, especially the paraventricular thalamic nucleus (PVT), a subregion of the thalamus (Fig. 4*A*,*B*,*D*), and the changes in c-Fos expression were negatively correlated with the withdrawal threshold (Fig. 4*E*). Conversely, the c-Fos expression in the OB_L brain region significantly decreased (Fig. 4*F*,*G*,*I*) and the changes in c-Fos expression were positively correlated with the withdrawal threshold (Fig. 4*J*). These findings consistent with the integrated ALFF and ReHo results from our cross-sectional and longitudinal fMRI analyses. Subsequently, we employed a potent opioid analgesic, morphine, to explore the activity changes in key brain regions (Th_L and OB_L) following the relief of neuropathic pain. This allowed us to further confirm the direct relationship between brain region activity changes and pain behavior. The results demonstrated that morphine effectively alleviated the enhanced c-Fos expression in the PVT caused by SNI (Fig. 4*A–D*). However, morphine treatment did not rectify the downregulation of c-Fos expression in the OB_L region induced by SNI, instead further exacerbating the decreasing trend (Fig. 4*F–I*). Correlation analysis revealed that the changes in c-Fos expression were still negatively correlated with the withdrawal threshold of the rats (Fig. 4*E*). However, there is no correlation between c-Fos changes in the OB_L brain region and withdrawal threshold (Fig. 4*J*).

### SNI leads to the change of FC of ROI to the whole brain in rats

**Figure 5.**
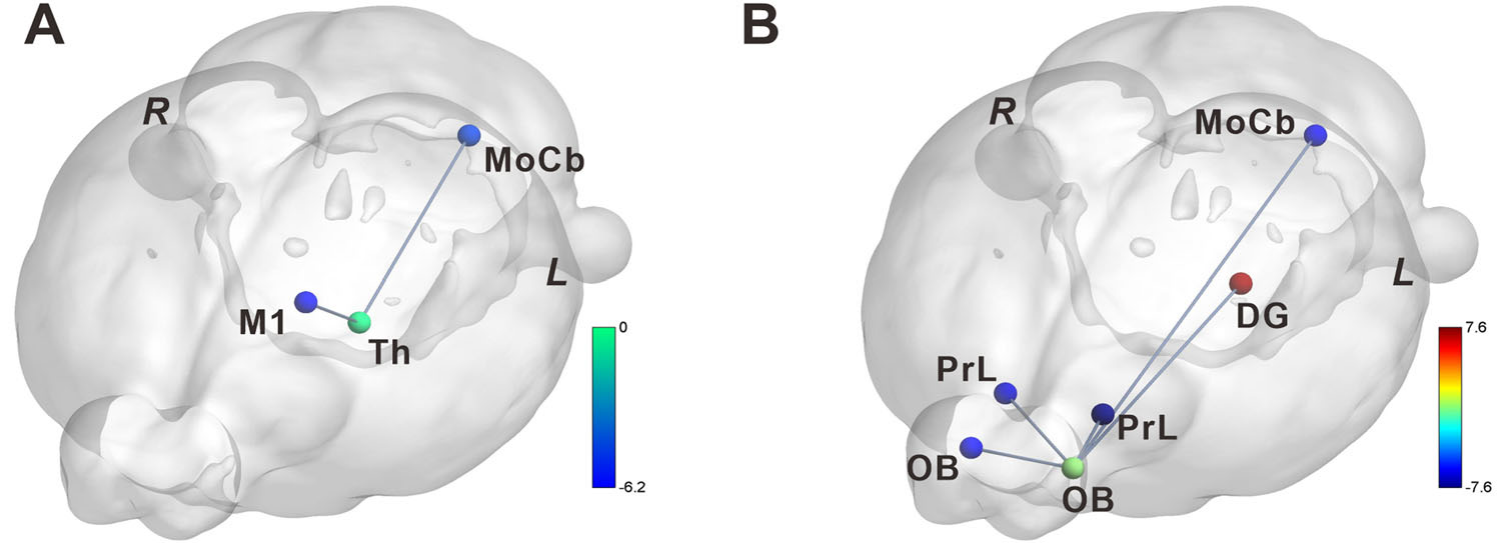
SNI-induced changes of functional connectivity in specific brain regions. ***A***, FC from TH_L (a intersection of cross-sectional and longitudinal studies) to whole brain regions of rats (bar in the right: *t* value of cluster; blue represents decrease; *n* = 11 in sham group and *n* = 10 in SNI group for cross-sectional study; *n* = 11 for longitudinal study). ***B***, FC from OB_L (a intersection of cross-sectional and longitudinal studies) to whole brain regions of rats in longitudinal study (bar in the right: *t* value of cluster; blue represents decrease and red represents increase; *n* = 11 in sham group and *n* = 10 in SNI group for cross-sectional study; *n* = 11 for longitudinal study). R, right; L, left.

The ROIs identified through BOLD-fMRI signal and correlation analysis were used as seed points (Th_L and OB_L) to investigate the SNI-induced alterations in FC associated with whole brain regions in rats. The results showed the major decreased alterations in FC with the Th_L occurred in the left molecular layer of the cerebellum (MoCb_L) and left Primary Motor Cortex (M1_L; Fig. 5*A* and Table 5). The major increase alterations in FC with the OB_L occurred in the left dentate gyrus (DG_L), and the decreased alterations with the OB_L occurred in the right olfactory bulb (OB_R), left prelimbic system (PrL_L), right prelimbic system (PrL_R), and MoCb_L (Fig. 5*B* and Table 6).

**Table 5.** Results of FC analysis of left thalamus to the whole brain

	Brain regions	MNI coordinates			Voxel size	Peak
		*x*	*y*	*z*		
Cross-sectional study						
Pain < HC	Molecular layer of the cerebellum L	−56	−77.05	57.2	696	−4.967
	Thalamus R	28	−17.05	18.2	180	−3.922
	Primary motor cortex L	−32	6.95	24.2	66	−3.5889
Longitudinal study						
Pain > HC	Primary auditory cortex R	70	−41.05	15.2	180	7.3898
Pain < HC	Perirhinal area 36 L	−59	−53.05	−26.8	145	−5.5106
	Molecular layer of the cerebellum L	−32	−137.05	−29.8	333	−4.8212
	Primary motor cortex L	−20	66.95	18.2	73	−6.148

**Table 6.** Results of FC analysis of left olfactory bulb to the whole brain

	Brain regions	MNI coordinates			Voxel size	Peak
		*x*	*y*	*z*		
Cross-sectional study						
Pain > HC	Dentate gyrus L	−68	−83.05	0.199997	10	4.4876
Pain < HC	Prelimbic system R	10	33.95	−17.8	82	−4.7883
	Molecular layer of the cerebellum L	40	−116.05	39.2	559	−6.0063
	Prelimbic system L	−41	27.95	30.2	98	−4.9594
	Olfactory bulb R	−8	96.95	33.2	295	−5.5237
Longitudinal study						
Pain > HC	Dentate gyrus L	−20	−2.05	24.2	80	7.0626
	Dorsolateral orbital cortex R	37	54.95	27.2	61	9.4436
Pain < HC	Brainstem L	−35	−80.05	0.199997	69	−5.0537
	Molecular layer of the cerebellum L	−35	−137.05	−2.800003	260	−6.153
	Prelimbic system L	−26	39.95	12.2	191	−7.5978
	Olfactory bulb R	16	30.95	−5.8	60	−5.8572
	Prelimbic system R	7	45.95	33.2	138	−6.5099

**Figure 6.**
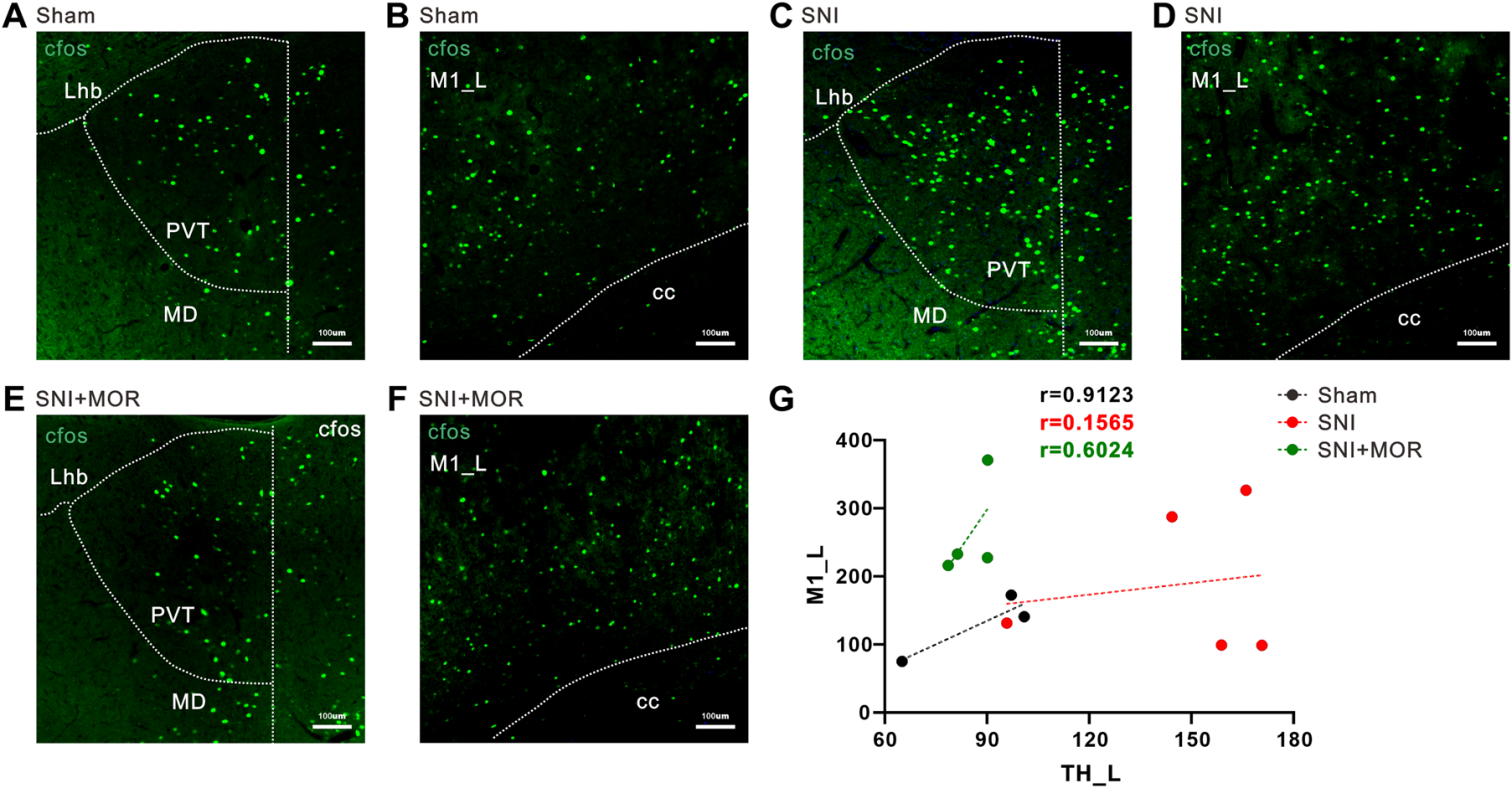
The correlation between changes in c-Fos expression across brain regions. ***A–F***, Representative immunofluorescence images of c-*fos* expression in the sham, SNI, and SNI + MOR group (scale bar, 100 μm). ***G***, Correlations between changes in c-*fos* numbers of PVT_L and M1_L (sham group, *n* = 3, black icon; SNI group, *n* = 5, red icon; SNI + MOR group, *n* = 4, green icon).

**Figure 7.**
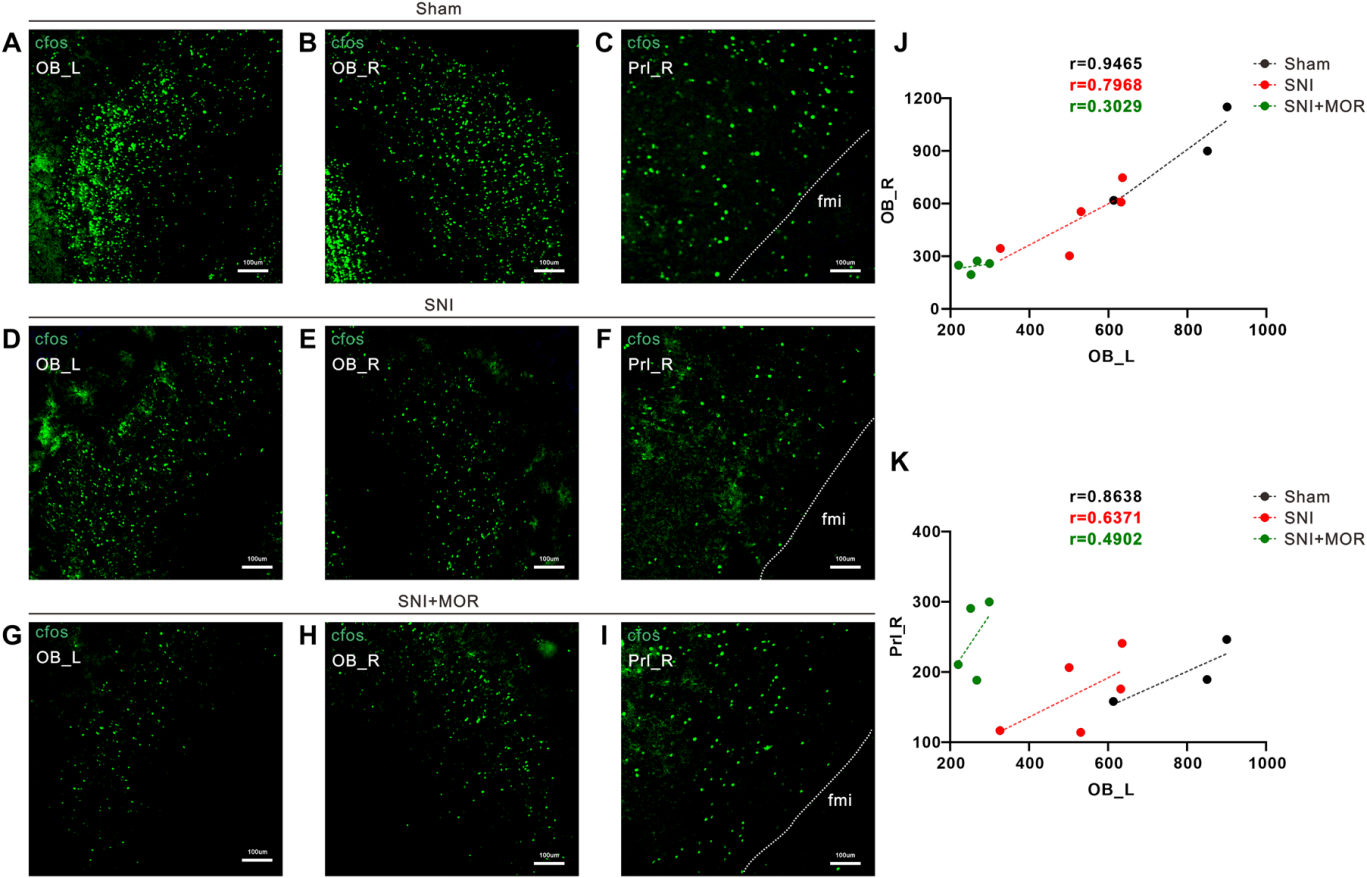
The correlation between changes in c-Fos expression across brain regions. ***A–I***, Representative immunofluorescence images of c-*fos* expression in the sham, SNI, and SNI + MOR group (scale bar, 100 μm). ***J***, ***K***, Correlations between changes in c-*fos* numbers of OB_L and OB_R, OB_L and Prl_R, respectively (sham group, *n* = 3, black icon; SNI group, *n* = 5, red icon; SNI + MOR group, *n* = 4, green icon).

To validate the results above, we performed c-Fos staining on whole-brain sections of rats, with a particular focus on calculating the correlation between c-Fos expression in brain regions showing FC changes identified by fMRI. The correlation analysis results provided evidence for the synchronous decrease in activity (decreased in FC values) of TH_L and M1_L in the SNI group (Fig. 6*A–D*,*G*). Furthermore, the administration of morphine for pain relief corrected the observed synchronous decrease in activity (Fig. 6*A–G*). In addition, correlation analysis also confirmed the decreased FC between OB_L-OB_R and OB_L-PrL_R observed in the fMRI results (Fig. 7*A–F*,*J*,*K*). However, the administration of morphine for pain relief did not reverse the observed changes in interregional connectivity caused by pain (Fig. 7*A–K*). These findings suggest that the activity changes in the OB_L brain region and accompanying alterations in interregional connectivity induced by SNI may not necessarily be associated with the analgesic effects of morphine.

## Discussion

Deeply exploring and understanding the adaptive activity and FC changes that occur in the brain during neuropathic pain can provide a potential basis for us to gain a comprehensive understanding of disease and further seek reasonable management methods. In the present study, we aimed to obtain more convincing conclusions by cross-analyzing the results from cross-sectional and longitudinal studies, combined with the use of analgesic drugs and the whole-brain c-Fos staining results, cautiously excluding potential confounding factors, in order to propose brain regions and FC changes that are more closely associated with nerve injury-induced chronic pain. Firstly, we performed ALFF and ReHo analyses on the signals captured by a multiple 9.4 T MRI machine, calculating the intersection brain regions where changes in activity and regional coherence occurred after SNI in both cross-sectional and longitudinal studies, thus identifying two significantly altered brain regions—Th_L (increase) and OB_L (decrease). The ALFF and ReHo values of imaging data indeed correlated with the mechanical painful withdrawal threshold. The c-Fos staining of rat brain tissue sections further confirmed the results of ALFF and ReHo. However, after the use of morphine analgesia, SNI-induced changes in activity in TH_L were almost corrected, while OB showed more inhibition of activity. Finally, we used these two key brain regions as seed points to conduct FC analysis toward the whole brain. The results also verified by whole-brain c-Fos staining mainly observed a significant decrease in connectivity between Th_L and M1_L after SNI treatment, and this decrease was corrected by morphine analgesia. OB_L exhibited decreased connectivity with the OB_R and PrL_R following SNI, but this decrease could not be relieved by morphine. Taken together, these results suggest that the thalamus is likely to be the most important target for modulating neuropathic pain.

The thalamus (Th), also known as the dorsal thalamus, is the largest ovoid gray nuclear mass in the diencephalon. Based on the anatomical location and incoming and outgoing connections of the thalamus, it can be divided into seven subnuclei groups, namely, anterior, lateral, ventral, medial, intralaminar, posterior nuclear groups, and the reticular nucleus (Boelens Keun et al., 2021). Previous studies have shown from various perspectives that the thalamus is the most important integration center for sensory information (except for olfaction) before it reaches the subjective perception of the cerebral cortex. The thalamus ventral group, intralaminar group, and posterior group receive fiber projections from the spinal cord and brainstem and relay them to the cerebral cortex through their posterior projections (Kawakita et al., 1993; Song et al., 2021; Al-Chalabi et al., 2023). In human fMRI studies, the thalamus is one of the brain regions most consistently activated by painful stimuli (Mercer Lindsay et al., 2021). Moreover, clinical studies have shown that patients with postherpetic neuralgia and low back pain exhibit alterations in the structure and volume of the thalamus (Li et al., 2020, 2021). In this study, although we did not perform structural imaging fMRI data analysis, we have confirmed significant changes in the activity and function of the thalamus during the course of neuropathic pain in rat models through the integration of cross-sectional and longitudinal research. On the basis of the imaging data and with reference to methods that have been used in the literature (Vetere et al., 2017; Roland et al., 2023), we also verified the changes in brain region activity and FC by immunofluorescence experiments on brain tissue sections. Changes in the above results after the use of drugs for pain relief were also observed, providing convincing results. Additionally, it is worth noting that in our experiment, SNI was performed on the left hindlimb of rats, and fMRI results showed a significant increase in ALFF and ReHo values in the ipsilateral (left) thalamus after the operation. This may seem contrary to what we have learned in textbooks, but in the current era of rapid development in neuroscience research methods and theories, it can be understood and explained. Possible underlying mechanisms include the ascending pathway for pain transmission from the spinal cord can result in bilateral projection upon reaching the brain, and at all levels of processing, the nociceptive message is modulated, amplified, or inhibited (Hylden et al., 1989; Price et al., 2003; Deng et al., 2020). Finally, our study also revealed a decreased FC between the Th_L and the MoCb_L and the M1_L, indicating a state of decoupling. c-Fos staining and correlation analysis verified the changes of connectivity. These findings may provide a novel perspective for understanding the neuropathic pain, and the precise neural circuits and functions involved require further analysis with additional neural circuit markers and functional experiments in the future.

The olfactory bulb (OB), as the first olfactory relay station in the brain, is known to heavily process sensory information. To adapt to an animal's needs, OB activity can be influenced by many factors either from within (intrinsic neuromodulation) or outside (extrinsic neuromodulation) the OB which include neurotransmitters, neuromodulators, hormones, and neuropeptides (Brunert and Rothermel, 2021). Moreover, recent studies demonstrate that the role of the OB is not limited to the olfactory system, but that it impacts many brain functions related to multiple disease, including neurodegeneration in aging, neurological, and neuropsychiatric disorders (Bhatia-Dey and Heinbockel, 2021; Delavari et al., 2021; Kesserwani, 2021). Our research results also clearly demonstrated a significant decrease in the BOLD signals of the OB_L following SNI-induced neuropathic pain. This suggests that the olfactory system may be an important functional gateway for understanding and analyzing neural regulation of pathological pain. In the present study, we have found that there is indeed a relationship between the activity of the OB and the changes in FC and the pain threshold in rats induced by nerve injury. However, when the analgesic drug morphine is introduced to suppress pain, the correlation between the two is disrupted—indicating that morphine cannot correct the brain activity and FC changes induced by neuropathic pain. The reason for this may be the low expression density of opioid receptors in the olfactory bulb region. Additionally, this suggests that there may be some receptors distributed in the olfactory bulb that mediate tolerance or exacerbate pain caused by morphine. In future research, we can focus on investigating whether olfactory dysfunction and defects may be a prodromal symptom of neuropathic pain and delve deeper into the potential neural mechanisms involved.

Additionally, we also observed that in both cross-sectional and longitudinal studies, there are some brain regions, such as periaqueductal gray (PAG) and retrosplenial dysgranular cortex (RSD), that show significant differences only in specific comparisons. We cannot simply dismiss the adaptive changes in these regions and their relationship with neuropathic pain. Instead, in future studies, it is important to carefully investigate the functionality of these regions by increasing sample size and integrating results from different institutions to mitigate differences arising from interindividual variability in cross-sectional studies. In longitudinal studies, inclusion of control groups and multiple time points can help eliminate differences observed at specific time points. These approaches will enhance the reliability and robustness of the findings.

### Conclusion

Our article revealed significant changes in brain activity and functional network connectivity in rats following nerve injury through the simultaneous implementation of both cross-sectional and longitudinal research. The results suggested that the thalamus and olfactory bulb were the key functional brain regions that were involved in SNI-induced mechanical hypersensitivity. It provides reliable ideas and directions for further exploring the brain network and neural circuit mechanisms of neuropathic pain.

## Synthesis

Reviewing Editor: Julie Bakker, University of Liege Decisions are customarily a result of the Reviewing Editor and the peer reviewers coming together and discussing their recommendations until a consensus is reached. When revisions are invited, a fact-based synthesis statement explaining their decision and outlining what is needed to prepare a revision will be listed below. The following reviewer(s) agreed to reveal their identity: Joanes Grandjean.

Synthesis: the revision has been seen by one reviewer who is positive, see commments below:

Reviewer 1

I thank the authors for considering my comments. The addition of cfos immunohistochemistry really strengthen the authors' claims.

I congratulate the authors for their study.
